# Mapping the subcellular pharmacokinetic-pharmacodynamic landscape: a systems-pharmacology framework for predicting combination therapies against intracellular bacterial infections

**DOI:** 10.3389/fphar.2026.1994202

**Published:** 2026-10-22

**Authors:** Venkata Bharat Kumar Pinnelli, Surendra Babu Thangachi, C. A. Jayashankar, Venkataramana Kandi, G. R. Manish, Koshy T. Sam, Prafulla Kumar Raju Bhupathi Raju, Atul Srinivas Sucharitha, Ankita Barman, G. Sainath, Kabir Singal

**Affiliations:** 1 Department of Biochemistry, Vydehi Institute of Medical Sciences and Research Centre, Bengaluru, Karnataka, India; 2 Department of Anatomy, Vydehi Institute of Medical Sciences and Research Centre, Bengaluru, Karnataka, India; 3 Department of General Medicine, Vydehi Institute of Medical Sciences and Research Centre, Bengaluru, Karnataka, India; 4 Department of Microbiology, Prathima Institute of Medical Sciences, Karimnagar, Telangana, India; 5 Department of General Medicine, Akash Institute of Medical Sciences and Research Centre, Bengaluru, Karnataka, India

**Keywords:** subcellular pharmacokinetics, pharmacodynamics, intracellular pathogens, systems pharmacology, drug synergy, host-directed therapy, antimicrobial resistance

## Abstract

Intracellular bacterial pathogens represent one of the most formidable challenges in contemporary medicine. By exploiting host cellular niches, these organisms evade immune surveillance and withstand conventional antibiotic therapies, establishing persistent reservoirs that are difficult to eradicate. Although antibiotic combinations, host-directed interventions, and drug repurposing have shown promise, conventional discovery pipelines rarely capture the dynamic and compartmentalized nature of the intracellular environment. This review proposes a systems pharmacology framework centered on subcellular pharmacokinetic/pharmacodynamic (Sub-PK/PD) profiling to predict and optimize intracellular combination therapies. Mechanistic and translational studies of intracellular drug disposition, host–pathogen metabolism, computational combination design, and biomimetic infection models were synthesized in a targeted narrative review. Particular attention was given to spatially resolved measurements, dual ribonucleic acid (RNA) sequencing, metabolic flux analysis, machine learning, and organ-on-a-chip platforms. Pathogen-containing vacuoles, phagolysosomes, the host cytosol, lipid-rich macrophages, and tissue lesions impose distinct barriers to drug ionization, accumulation, stability, and target engagement. Persistent infection also produces reciprocal metabolic rewiring: host pathways supply carbon, nitrogen, lipids, metals, and reducing equivalents, while bacterial stress programs alter growth and antibiotic susceptibility. Integrating these processes can reveal shared metabolic bottlenecks suitable for combinations of pathogen-directed and host-directed agents. Computational models can prioritize combinations, but their predictions require validation under physiologically realistic concentration–time profiles. Linking systems biology to localized Sub-PK/PD provides a practical roadmap for designing niche-specific regimens. Such regimens could improve clearance of persistent intracellular reservoirs, reduce ineffective exposure, and limit the emergence of antimicrobial resistance.

## 1 Introduction

Intracellular survival is a convergent strategy used by phylogenetically diverse bacteria. *Mycobacterium tuberculosis* persists in macrophage phagosomes and heterogeneous pulmonary lesions; *Salmonella enterica* remodels a membrane-bound vacuole while a subpopulation enters a nonreplicating state; *Listeria monocytogenes* escapes into the cytosol; *Chlamydia* species construct specialized inclusions that intercept host lipids; and uropathogenic *Escherichia coli* (UPEC) can form intracellular bacterial communities in bladder epithelium (Hackstadt et al., 1995; Scidmore et al., 1996; Carabeo et al., 2003; Anderson et al., 2003; Mysorekar and Hultgren, 2006). These niches shield organisms from antibodies and complement, reshape innate immune sensing, and expose bacteria to nutrient limitation, low pH, reactive species, hypoxia, and metal stress. The resulting populations are heterogeneous in location, replication rate, and metabolic activity.

The intracellular phase may be obligate, facultative, or transient, but in each case, it can shape disease chronicity and treatment outcome. Infected phagocytes can disseminate organisms, epithelial reservoirs can seed recurrent mucosal infection, and long-lived host cells can preserve viable bacteria after symptoms improve. Intracellular bacterial communities and distinct reservoir populations have also been detected in human or experimental urinary tract infection (Rosen et al., 2007; Schwartz et al., 2011). The consequences include prolonged therapy, relapse after apparently adequate treatment, and reduced efficacy at sites where immune access and drug penetration are limited. Intracellular persistence is therefore a broad therapeutic obstacle spanning respiratory, enteric, genitourinary, and systemic infections, particularly when cellular immunity is impaired.

Genetic resistance must be distinguished from phenotypic tolerance and persistence. Resistance raises the concentration needed to inhibit growth through heritable mechanisms such as target modification, drug inactivation, or altered transport. Tolerance enables a susceptible population to survive longer exposure without changing the minimum inhibitory concentration, while persistence describes a small, reversible subpopulation that survives otherwise lethal treatment. Single-cell and evolution experiments show that persistence can function as a phenotypic switch and that prolonged survival under antibiotics facilitates subsequent resistance evolution (Balaban et al., 2004; Levin-Reisman et al., 2017; Windels et al., 2019). Intracellular stress can encourage each outcome indirectly: limited exposure favors resistant mutants, slow growth weakens replication-dependent antibiotics, and heterogeneous host cells create protected populations. Effective regimens must therefore address both inherited resistance and the nonheritable states that permit rebound.

This heterogeneity creates a pharmacological problem as much as a microbiological one. Plasma or tissue drug concentration does not establish that the unbound, active compound reaches the bacterium. A drug must cross tissue barriers, enter the infected cell, avoid efflux or inactivation, partition into the correct organelle, and remain active under the local physicochemical conditions. Studies using imaging mass spectrometry, fluorescent analogues, and subcellular fractionation demonstrate that antimicrobial agents with similar plasma exposure can distribute very differently across lesions, immune-cell types, and intracellular compartments (Prideaux et al., 2015; Schump et al., 2017; Blanc et al., 2018; Greenwood et al., 2019).

Conventional pharmacokinetic indices can therefore misrepresent the exposure that drives intracellular killing. A high plasma area under the concentration–time curve may coexist with poor cellular uptake, while extensive total-cell accumulation may reflect membrane binding or sequestration away from the pathogen. Comparative macrophage studies demonstrate substantial differences among antibiotic classes and show that extracellular and intracellular concentration–response relationships cannot be assumed to be equivalent (Carryn et al., 2002; Seral et al., 2005; Barcia-Macay et al., 2006; Lemaire et al., 2011). Lipophilicity, charge, ionization, and protein binding interact with transporters, organelle pH, and host metabolism. The same drug may thus reach different effective concentrations in macrophages, epithelial cells, and neutrophils. Local conditions also change bacterial growth, target abundance, membrane potential, and drug stability. The central question is not merely whether a compound enters cells, but where its active fraction resides over time.

Persistence further separates conventional susceptibility testing from therapeutic response. Macrophage uptake can induce nonreplicating *Salmonella* persisters, while the intracellular environment can activate bacterial efflux and stress programs that produce reversible antibiotic tolerance (Adams et al., 2011; Helaine et al., 2014; Stapels et al., 2018). Dynamic single-cell studies in mycobacteria, intracellular *Staphylococcus aureus*, and UPEC further demonstrate survival heterogeneity during antimicrobial exposure (Wakamoto et al., 2013; Peyrusson et al., 2020; Blango and Mulvey, 2010). These phenotypes do not necessarily involve stable resistance mutations, yet they prolong infection and create opportunities for genetic resistance when effective monotherapy occurs in a protected niche.

Combination therapy is a logical response, but adding active drugs does not guarantee complementary intracellular action. Partners that are synergistic in nutrient-rich broth may become indifferent or antagonistic in acidic, hypoxic, or nutrient-limited compartments, and antichlamydial activity can vary across the bacterial developmental cycle (Siewert et al., 2005). Differences in penetration, organelle partitioning, or half-life can create intervals in which only one agent is effective. Host-directed agents add complexity because their effects may depend on immune phenotype, infection stage, and dosing order. Rational combinations must therefore align mechanism, location, bacterial state, and time, especially when repurposed drugs were not developed to achieve anti-infective subcellular exposure.

Recent experimental and computational methods make this alignment feasible. Spatial mass spectrometry and live-cell imaging can map drug distribution; dual and single-cell RNA sequencing can resolve host and bacterial responses; isotope tracing and constraint-based models can identify nutrient dependencies; and machine learning can prioritize combinations. Organoids and organ-on-a-chip (OOC) systems can test predictions under tissue architecture, flow, and dynamic dosing. Yet these approaches are often disconnected: localization studies may omit bacterial physiology, omics may ignore achievable exposure, and computational predictions may be validated only in static monolayers. A unifying framework must connect molecular hypotheses to localized drug action and translational models.

The central argument of this review is that rational combination therapy should be designed at the scale at which the drugs and organisms interact. We term this framework subcellular pharmacokinetics/pharmacodynamics (Sub-PK/PD). Sub-PK describes the concentration–time profile of active drug at a defined intracellular site, whereas Sub-PD describes how bacteria in that site respond as a function of physiological state and host context. The framework joins four layers:

1. Spatial drug delivery
2. Host–pathogen metabolic coupling
3. Computational selection of complementary interventions
4. Validation in models that reproduce human tissue architecture and temporal dosing.

## 2 Literature selection and conceptual scope

This review was conceived as a targeted narrative synthesis and conceptual framework rather than a systematic review or meta-analysis. The rationale for this design lies in the heterogeneity of the evidence base, which spans cellular microbiology, clinical pharmacology, constraint-based systems biology, and microfluidic engineering. The primary objective is to integrate domains that are often examined in isolation, such as subcellular drug disposition, phenotypic bacterial tolerance, host–pathogen metabolic coupling, computational prediction of drug combinations, and biomimetic microphysiological systems, into a practical Sub-PK/PD roadmap for rational regimen selection. The evidence base was compiled from PubMed-indexed primary literature published through mid-2026, supplemented by reference chaining and targeted queries addressing spatial pharmacology, paired dual RNA-seq transcriptomics, metabolic flux modeling, machine learning, and organ-on-a-chip technologies. Searches iteratively combined pathogen/compartment descriptors (phagosome, phagolysosome, vacuole, cytosol) with pharmacological and computational metrics (ion trapping, time-kill kinetics, persistence, metabolic flux analysis, graph learning). Studies were selected based on their mechanistic relevance to bacterial pathogens exhibiting obligate, facultative, or transient intracellular niches (such as *M. tuberculosis*, *S. enterica*, *L. monocytogenes*, *Chlamydia* spp., and uropathogenic *E. coli*). Priority was assigned to investigations providing quantitative concentration–response data, direct subcellular spatial resolution, dynamic killing kinetics, paired host-pathogen perturbation, or microphysiological validation. Because the underlying literature encompasses analytical mass spectrometry, non-coding ribonucleic acid (RNA) profiling, stoichiometric computational simulations, and micro-engineered devices, a single formal risk-of-bias instrument was inapplicable. Evidence was instead appraised through domain-specific criteria: spatial resolution and unbound-versus-total drug verification for imaging studies; viable-count confirmation and extracellular controls for infection assays; biological replication and external validation for machine-learning architectures; and physiological shear and barrier differentiation for microphysiological systems. While conceptual selection carries inherent publication bias toward positive findings and well-studied model organisms, this framework synthesizes recurring systems-level mechanisms into testable hypotheses rather than an empirical ranking of clinical regimens.

## 3 Intracellular infection as a spatial pharmacology problem

### 3.1 The tissue-to-bacterium concentration cascade

For an extracellular infection, unbound drug in interstitial fluid may approximate the concentration available to the organism. For an intracellular infection, exposure is a cascade. A useful abstraction is

$$C_{\text{niche}}\left(t\right)=f\;C_{\text{plasma},u}\left(t\right),P_{\text{tissue}},P_{\text{cell}},pK_{a},\text{pH},T_{\text{in}/\text{out}},B,M,S$$

where *C*_plasma,u_ is unbound plasma concentration; *P*_tissue_ and *P*_cell_ represent tissue and cellular permeability; *T*_in/out_ represents influx and efflux transport; *B* represents binding; *M* represents metabolism; and *S* represents sequestration. Each term may vary with cell type, activation state, organelle maturation, and infection time. Total cellular concentration can therefore be misleading: a high value may reflect inactive sequestration in lysosomes or membranes rather than exposure at the bacterial surface (Figure 1).

**FIGURE 1:**
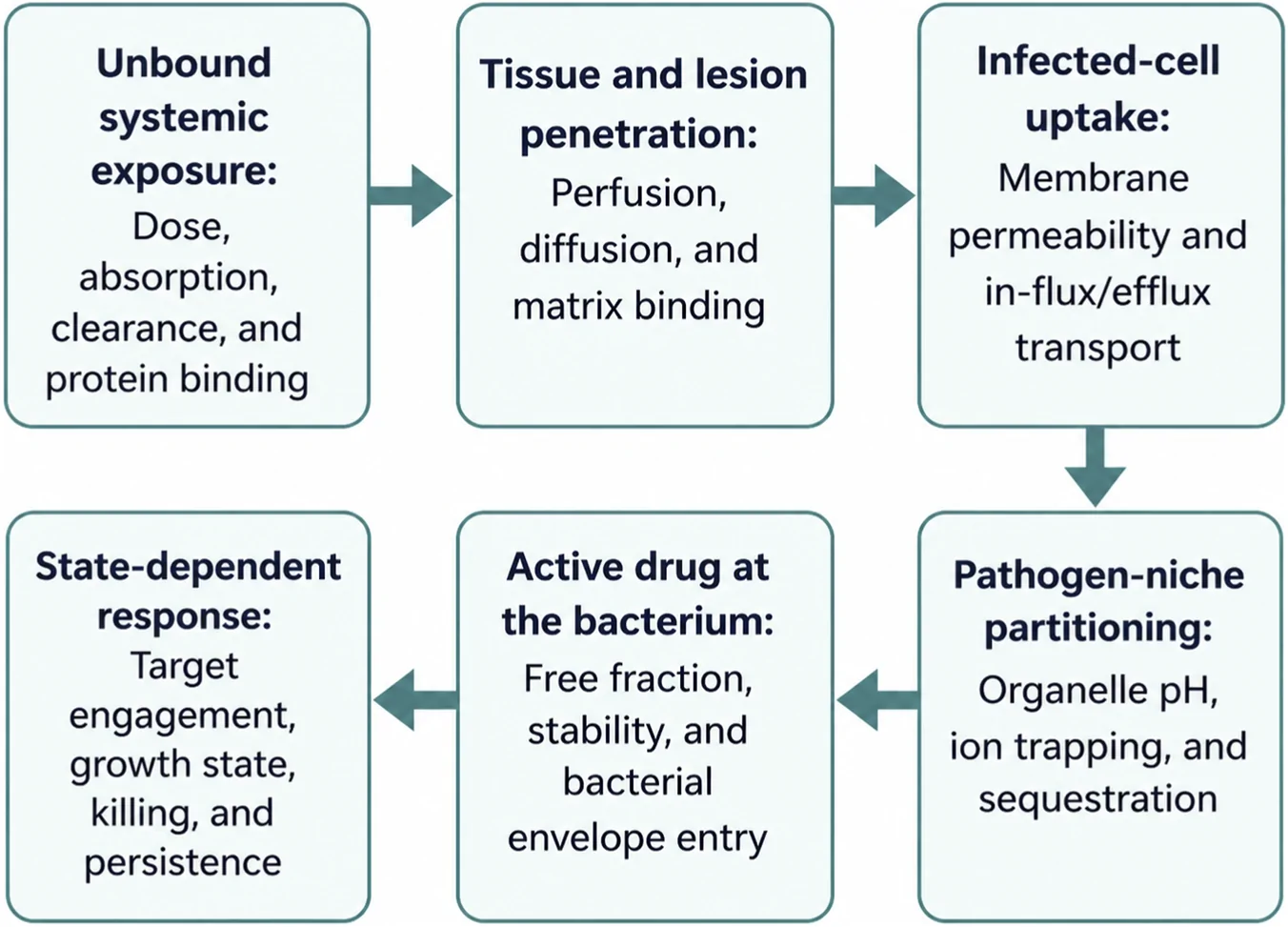
The Sub-PK/PD exposure cascade. Systemic exposure is filtered through sequential tissue, cellular, and organelle barriers before producing a bacterial response. Failure at any step can uncouple plasma pharmacokinetics from intracellular efficacy.

The importance of spatial distribution is well demonstrated in tuberculosis. Drug penetration differs between cellular rims and avascular caseum, and sterilizing activity is associated with whether adequate concentrations reach the lesion compartments that contain persistent bacilli (Prideaux et al., 2015; Sarathy et al., 2018). High-resolution imaging further shows preferential fluoroquinolone accumulation in particular immune-cell populations (Blanc et al., 2018). These lesion-scale observations are directly relevant to intracellular therapy because the infected cell is embedded within a larger anatomical barrier system.

### 3.2 Ionization, pH partitioning, and organelle trapping

Weak bases often accumulate in acidic lysosomes through ion trapping: the uncharged fraction crosses the membrane, becomes protonated in the lumen, and is less able to diffuse back. This mechanism can produce cellular concentrations many times greater than extracellular concentrations. However, accumulation is not equivalent to activity. Acidic pH may reduce bacterial uptake, alter target binding, or destabilize a compound. Conversely, drugs such as pyrazinamide exploit acidic or stressed environments and may display activity not predicted by neutral broth assays.

Subcellular partitioning studies of antitubercular agents show that physicochemical properties influence whether compounds concentrate in lipid droplets, acidic compartments, membranes, or cytosol, and that intramacrophage selectivity can diverge from axenic potency (Schump et al., 2017). Direct visualization has also revealed intracellular drug reservoirs that exchange dynamically with the surrounding medium and infected compartments (Greenwood et al., 2019). Thus, a Sub-PK assay should distinguish at least extracellular medium, cytosol, pathogen-containing vacuole, lysosome, and membrane- or lipid-associated fractions.

### 3.3 Pathogen-containing vacuoles and the host cytosol

Not all membrane-bound niches are equivalent. The mycobacterial phagosome undergoes restricted maturation; the *Salmonella*-containing vacuole recruits host trafficking machinery and develops tubular extensions; and the chlamydial inclusion intercepts lipids and vesicles without becoming a conventional lysosome. Their luminal pH, ion composition, protease activity, and membrane transport differ. A delivery strategy optimized for terminal lysosomes may therefore fail against bacteria in a less acidic, pathogen-remodeled vacuole.

Cytosolic pathogens present the opposite problem. Agents that accumulate almost exclusively in acidic organelles may have low free cytosolic exposure. Cytosolic activity also depends on nonspecific protein and membrane binding, host metabolism, and access across the bacterial envelope. Combination partners should therefore be assessed for spatial complementarity: either both agents co-localize with the same bacterial population, or each intentionally covers a different population.

### 3.4 Subcellular pharmacodynamics and phenotypic tolerance

Sub-PD extends the usual minimum inhibitory concentration by conditioning response on niche and bacterial state. Intracellular bacteria may be rapidly replicating, slow-growing, nonreplicating but metabolically active, or deeply dormant. Macrophage internalization induces a nonreplicating *Salmonella* persister fraction, and those persisters can reshape immune responses during therapy (Helaine et al., 2014; Stapels et al., 2018). In mycobacteria, macrophage-induced efflux contributes to reversible drug tolerance (Adams et al., 2011). Caseous material likewise supports extreme phenotypic tolerance that is incompletely reproduced in standard broth (Sarathy et al., 2018).

An informative intracellular time-kill experiment should therefore report the full viable-count trajectory, post-antibiotic regrowth, host-cell viability, and bacterial state markers. Static fold reduction at a single time point can confuse delayed growth with killing. For combinations, the relevant question is whether concurrent niche exposure produces faster killing, a lower persister fraction, or suppression of regrowth without unacceptable host toxicity (Table 1).

**TABLE 1:** Representative intracellular and lesion-associated niches and their implications for combination design.

Niche	Dominant features	Sub-PK/PD risk	Design implication
Acidic phagolysosome	Low pH, hydrolases, reactive species, cation accumulation	Ion trapping without target engagement; pH-dependent instability or activity loss	Measure unbound luminal exposure and potency at matched pH; pair delivery with a stress-active agent
Pathogen-remodeled vacuole	Altered maturation, nutrient transport, variable membrane permeability	Lysosome-targeted compounds may miss the compartment	Quantify compartment-specific localization and trafficking over time
Host cytosol	Near-neutral pH, high protein content, host metabolic enzymes	Low free exposure despite high total-cell drug; rapid metabolism	Prioritize cytosol-available compounds and measure free intracellular fraction
Lipid-rich macrophage	Lipid droplets, foamy phenotype, altered membrane composition	Lipophilic sequestration; bacterial use of host lipids; slow growth	Combine a penetrating antibacterial with a safely validated lipid- or autophagy-modulating intervention
Necrotic lesion/caseum	Poor vascularization, debris binding, variable pH, nonreplicating bacilli	Incomplete penetration and extreme tolerance create spatial monotherapy	Select partners with complementary lesion penetration and activity against nonreplicating organisms

## 4 Host–pathogen metabolic cross-talk

### 4.1 Infection rewires both metabolic networks

The infected cell is not a passive container. Pattern-recognition signaling, cytokines, hypoxia, and organelle damage can alter glycolysis, oxidative phosphorylation, fatty-acid handling, amino-acid metabolism, redox balance, and metal transport. Bacterial effectors reinforce or redirect these changes. The pathogen then consumes metabolites generated or imported by the host, producing a coupled metabolic system in which neither organism can be understood in isolation.

The direction of host metabolic change is context dependent. Increased glycolysis can support antimicrobial functions in some activated macrophages, yet excessive or prolonged inflammation can damage tissue and impair effective immunity. Lipid accumulation may sequester inflammatory mediators, alter phagosome maturation, and provide carbon substrates. Consequently, a host pathway should not be labeled “protective” or “pathogenic” without defining cell type, infection stage, and organism.

### 4.2 Nutrient acquisition and bacterial persistence

Intracellular bacteria use specialized transporters and catabolic pathways to exploit restricted nutrients. *M. tuberculosis* requires host cholesterol utilization for persistence, can accumulate lipid inclusions in lipid-loaded macrophages, and consumes a mixed diet that includes amino acids and one- and two-carbon substrates (Pandey and Sassetti, 2008; Daniel et al., 2011; Beste et al., 2013). *Salmonella* likewise exploits multiple host nutrients in parallel rather than depending on a single carbon source (Steeb et al., 2013). This flexibility explains why blocking one bacterial carbon pathway may have limited effect unless the host supply network or compensatory routes are also considered.

Nutrient restriction can also generate tolerance rather than death. Reduced carbon flux, oxygen limitation, acidic stress, and redox imbalance may slow replication and diminish the effect of growth-dependent antibiotics. A rational metabolic combination should therefore avoid simply forcing bacteria into a more tolerant state. One partner may restrict a critical nutrient, while a second targets the stress response, respiratory adaptation, or maintenance pathway required for survival during starvation.

### 4.3 Dual RNA sequencing and single-cell resolution

Dual RNA sequencing measures host and pathogen transcripts from the same infected sample. In *Salmonella*-infected cells, this approach exposed regulatory small RNAs and coordinated host–pathogen programs that would be obscured by analyzing either species separately (Westermann et al., 2016). Related paired-transcriptome approaches have resolved mycobacterial adaptation inside macrophages and dendritic cells and infection-specific regulatory programs in tissue (Tailleux et al., 2008; Rienksma et al., 2015; Nuss et al., 2017). Single-cell studies further showed that bacterial phenotypic variation drives heterogeneous host responses and that macrophage polarization correlates with intracellular bacterial growth (Avraham et al., 2015; Saliba et al., 2016).

These observations have two consequences for therapy design. First, average expression can conceal the minority cell population that maintains persistence. Second, correlations across paired host and bacterial states can nominate cross-species bottlenecks. For example, increased expression of a host nutrient exporter coupled to induction of the cognate bacterial importer is stronger evidence of dependency than either signal alone. Spatial transcriptomics and imaging-based RNA methods can add the missing anatomical coordinate.

### 4.4 Flux balance analysis and isotope tracing

Transcript abundance is an imperfect proxy for metabolic flux. Genome-scale metabolic models impose stoichiometric and thermodynamic constraints, allowing prediction of feasible flux states and essential reactions under defined nutrient conditions. When host and bacterial models are coupled through an exchange compartment, simulations can test which transported metabolites support pathogen biomass or maintenance and which host perturbations reduce that support.

Stable-isotope tracing is essential for constraining these models. The mixed carbon sources used by intracellular *M. tuberculosis* were revealed through ^13^C flux analysis rather than expression alone (Beste et al., 2013). A robust workflow iterates between computation and experiment: dual transcriptomics identifies active modules, metabolomics measures pool sizes, isotope tracing estimates direction and magnitude of flux, and genetic or pharmacological perturbation tests causality.

### 4.5 Criteria for actionable shared bottlenecks

A shared metabolic bottleneck is attractive only when five conditions are met. It should be active in the clinically relevant niche, necessary across heterogeneous bacterial states, incompletely buffered by alternative pathways, pharmacologically accessible, and separable from essential host physiology. The best targets often lie at an interface: a host transporter feeding a restricted vacuolar nutrient, a bacterial transporter with limited redundancy, or a stress-maintenance reaction selectively required under intracellular conditions.

Selectivity must be demonstrated rather than assumed. Host metabolic inhibition can produce immunosuppression, mitochondrial toxicity, or organ-specific adverse effects. Conversely, a modest host perturbation may be sufficient when combined with a direct antibacterial. This favors partial, time-limited modulation over chronic pathway ablation.

## 5 Deconvoluting hyper-dimensional host-pathogen data: the imperative for *in Silico* frameworks

While dual RNA sequencing (dual RNA-seq), metabolomics, and ^13^C-metabolic flux analysis resolve paired phenotypic adaptations (Beste et al., 2013; Westermann et al., 2016), they precipitate a severe dimensionality barrier. Dual transcriptomics yields high-dimensional matrices across heterogeneous host-cell and bacterial populations (Table 2) (Avraham et al., 2015; Saliba et al., 2016), while metabolomic cross-talk uncovers simultaneous shifts across interwoven lipid, carbohydrate, and cofactor networks (Steeb et al., 2013).

**TABLE 2:** Complementary methods for mapping intracellular pharmacology and host–pathogen state.

Method	Primary measurement	Decision supported	Important limitation
Subcellular fractionation with liquid chromatography–tandem mass spectrometry (LC–MS/MS)	Drug abundance in enriched cytosolic, membrane, or organelle fractions	Determines whether cellular accumulation reflects exposure in intended compartment	Organelle disruption, cross-contamination, and frequent measurement of total rather than unbound drug
Imaging mass spectrometry	Label-free spatial distribution across tissue and cellular regions	Identifies lesion penetration and spatially protected bacterial reservoirs	Spatial resolution may not reach individual organelles; ionization varies by tissue matrix
Fluorescent drug imaging	Live or fixed-cell localization and exchange kinetics	Reveals co-localization, trafficking, retention, and release	Fluorophore attachment can alter charge, permeability, binding, or antibacterial activity
Dual or single-cell RNA sequencing	Paired host and pathogen transcriptional states	Identifies heterogeneous stress programs, transport systems, and candidate dependencies	Low bacterial RNA abundance and transcription–flux discordance
Stable-isotope tracing and flux modeling	Substrate use, pathway direction, and exchange flux	Tests whether a predicted nutrient bottleneck is active and nonredundant	Requires explicit model assumptions and carefully selected labeled substrates
Dynamic intracellular time-kill assay	Viable burden during exposure, washout, and regrowth	Distinguishes killing from growth arrest and measures persistence or relapse	Throughput is lower than endpoint screens and results depend strongly on the host model

This combinatorial explosion creates an intractable search space: empirically screening multi-drug combinations across concentrations, temporal sequences, and fluctuating intracellular states is experimentally and economically unfeasible (Brochado et al., 2018). Furthermore, static omics profiling alone cannot infer dynamic flux rewiring or anticipate non-intuitive metabolic bypasses that emerge under antibiotic pressure (Beste et al., 2013).

Bridging the divide between high-dimensional biological observations and rational combination design necessitates deterministic and data-driven computational engines.

### 5.1 Constraint-based metabolic modeling (e.g., flux balance analysis [FBA])

Genome-scale metabolic reconstructions integrate dual transcriptomic and proteomic boundaries into stoichiometric networks. Coupled host-pathogen FBA simulates how pharmacological perturbation of an enzymatic node in either organism dynamically alters substrate availability and forces the pathogen into non-viable or non-compensatory metabolic dead-ends (Beste et al., 2013; Rienksma et al., 2015).

### 5.2 Graph neural networks (GNNs) and multiscale machine learning

To prioritize combinations beyond linear pathways, host-pathogen biological networks are framed as heterogeneous, multi-relational graphs, wherein nodes denote host proteins, bacterial targets, metabolites, and drug scaffolds, interconnected by edges representing transport, regulatory influence, or biochemical conversion (Lv et al., 2022). Multiscale pipelines digesting these topological architectures can rank synergistic partner compounds and predict *in vivo* efficacy profiles before committing resources to experimental validation (Ma et al., 2019; Cicchese et al., 2021; Lv et al., 2022).

By formalizing these biological dependencies computationally, regimens can be designed to exploit specific vulnerabilities across distinct intracellular niches, giving rise to the mechanistic combination archetypes outlined in the succeeding sections.

## 6 Rational design of intracellular combination therapies

### 6.1 Antibiotic–antibiotic combinations

Traditional combinations are selected to broaden coverage, accelerate killing, and suppress resistance. Systematic studies show that interactions vary across species and can be enhanced by nonantibiotic partners, while chemogenomic models can nominate combinations from conserved or divergent response networks (Ejim et al., 2011; Chandrasekaran et al., 2016; Brochado et al., 2018). Sub-PK/PD adds spatial and temporal criteria. Each component should achieve an effective concentration in the intended niche, and the periods above the niche-specific pharmacodynamic threshold should overlap. A combination that is synergistic in broth can behave as functional monotherapy if one component does not enter infected cells or penetrates the compartment only after the other has fallen below its active concentration.

Physiological diversity argues for combinations that cover complementary states. One agent may kill replicating bacteria, while another targets respiration or maintenance in nonreplicating cells. However, state transitions matter: a bacteriostatic partner can antagonize a growth-dependent bactericide, and nutrient restriction can either sensitize organisms or deepen tolerance. Checkerboards should therefore be supplemented by time-kill matrices, sequential-exposure experiments, and regrowth assays under niche-matched conditions.

### 6.2 Host-directed and pathogen-directed combinations

Host-directed therapy can act through at least four mechanisms: improving delivery to the infected compartment, enhancing phagosome maturation or autophagy, restricting a host-derived nutrient, or rebalancing damaging inflammation. Genome-scale knockdown and multiparametric chemical screens have identified host survival factors and triggers of intracellular mycobacterial killing, providing proof that host pathways can be searched systematically (Jayaswal et al., 2010; Sundaramurthy et al., 2013; Stanley et al., 2014). Statins enhanced autophagy and phagosome maturation in experimental tuberculosis, while metformin improved host control in preclinical and observational components of an adjunct-therapy study (Parihar et al., 2014; Singhal et al., 2014). Modulating inflammatory pathways has also produced host-directed benefit, with experimental evidence that optimal intervention can depend on host genotype and the balance between interleukin-1 and type I interferon signaling (Tobin et al., 2012; Mayer-Barber et al., 2014).

These examples are mechanistic leads, not interchangeable clinical recommendations. Host response depends on pathogen, disease stage, comorbidity, genotype, and concomitant drugs. A host-directed partner should be advanced only with a biomarker that confirms target engagement and a safety window that remains acceptable when combined with antibiotics.

### 6.3 Drug repurposing

Repurposing can shorten early development because human pharmacology and safety information already exist. Yet plasma exposure appropriate for the original indication may not yield the required subcellular exposure. Protein binding, lysosomal trapping, active metabolites, transporter inhibition, and tissue accumulation must be reassessed in the infection context. The relevant comparison is not the approved dose versus an extracellular minimum inhibitory concentration, but the clinically attainable unbound niche concentration versus an intracellular exposure–response relationship.

Repurposing screens are most informative when conducted in infected primary cells with matched uninfected controls. Hits should be triaged into direct antibacterial, host-mediated, and nonspecific cytotoxic classes. Counter-screens should include mitochondrial function, cytokine release, cell-type specificity, and interactions with the intended antibiotic backbone.

### 6.4 Artificial intelligence and graph-based prediction

The combinatorial search space grows rapidly with the number of agents, doses, schedules, cell states, and niches. Machine-learning models can prioritize experiments by integrating chemical structure, drug-induced transcription, bacterial pathways, host networks, and known drug–drug interactions. Transcriptomic signatures have already predicted regulators of antitubercular drug synergy and ranked regimens associated with clinical efficacy, while a linked multiscale model combined interaction predictions with pharmacokinetics and granuloma behavior (Ma et al., 2019; Cicchese et al., 2021).

Graph methods are well suited to this problem because drugs, targets, pathways, metabolites, cell states, and compartments can be represented as typed nodes connected by mechanistic edges. A graph model can learn that two compounds are complementary because one disrupts host nutrient supply and the other targets the bacterial pathway that consumes that nutrient. Graph-learning approaches have been used to predict synergistic antibiotic combinations, although prospective intracellular validation remains limited (Lv et al., 2022).

Deep learning has also expanded antibacterial chemical space, exemplified by the discovery of halicin, an *Acinetobacter baumannii*-selective compound, and explainable models that identified new structural classes (Stokes et al., 2020; Liu et al., 2023; Wong et al., 2024). For intracellular combination design, predictive performance alone is insufficient. Models should report uncertainty, preserve interpretable links to biology, and be tested on pathogens, cell types, and chemical scaffolds excluded from training.

### 6.5 Scheduling is part of the combination

Simultaneous administration is not always optimal. A host-directed primer may need time to mature phagosomes before antibiotic exposure; a metabolic perturbation may first sensitize bacteria, whereas prolonged starvation could induce tolerance. Conversely, a rapidly bactericidal drug may reduce inflammatory stimulus before an immunomodulator is introduced. Dynamic models should therefore search over order and interval as well as dose.

A practical metric is joint target coverage: the fraction of time during which both agents exceed their niche-specific effective concentrations while host toxicity remains below threshold. This metric can be extended across multiple niches and weighted by the estimated contribution of each reservoir to relapse.

## 7 Biomimetic models for translation

### 7.1 Limitations of static monolayers

Two-dimensional immortalized-cell monolayers are useful for throughput but poorly reproduce cell differentiation, extracellular matrix, oxygen gradients, vascular delivery, mechanical forces, and immune-cell recruitment. Constant drug concentration in culture also eliminates the peaks, troughs, and delays created by human dosing. These omissions are particularly damaging for Sub-PK/PD because compartment maturation and drug partitioning change over time.

Primary cells, organoids, three-dimensional granulomas, and microphysiological systems should be viewed as a model ladder rather than competing replacements. Human granuloma and lung-tissue systems reproduce selected features of mycobacterial dormancy, host architecture, and drug response, while hydrogel models can support niche-specific efficacy testing (Kapoor et al., 2013; Braian et al., 2015; Gupta et al., 2025). Early screens can use scalable macrophage or epithelial assays; mechanistic hits can progress to primary cells and organoids; and a smaller set can be evaluated under flow, tissue interfaces, and clinically realistic pharmacokinetic profiles (Figure 2).

**FIGURE 2:**
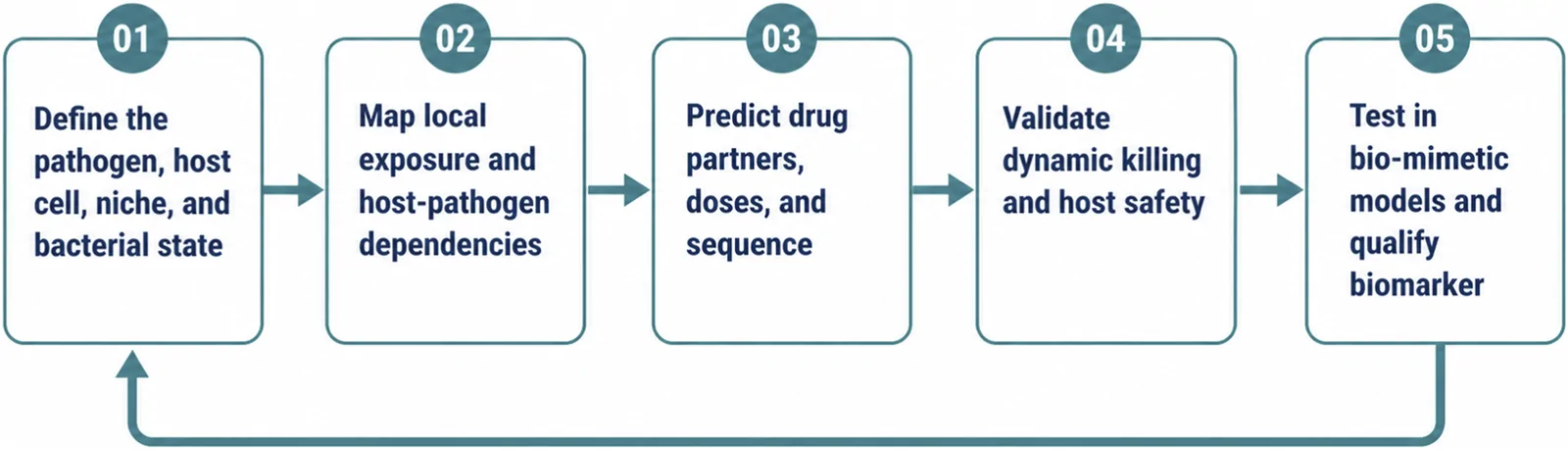
Closed-loop systems-pharmacology workflow for intracellular combination design. Experimental results feed back into target definition and model refinement rather than serving as a terminal validation step.

### 7.2 Organ-on-a-chip evidence

Microfluidic platforms can recreate epithelial–endothelial interfaces, shear stress, air or urine exposure, and controlled drug perfusion. A lung-on-a-chip model of early mycobacterial infection revealed a role for alveolar epithelial cells in restricting bacterial growth that was not apparent from macrophage-only models (Table 3) (Thacker et al., 2020). A human bladder-chip reproduced dynamic intracellular communities of uropathogenic *Escherichia coli*, demonstrating persistence under a differentiated, flowing tissue environment (Sharma et al., 2021). Three-dimensional microfluidic tuberculosis models have also been developed to study host responses and antimicrobial resistance in a human tissue-like context (Bielecka et al., 2017). Complementary organoid systems have modeled *Salmonella* interactions with intestinal epithelium and *Chlamydia* infection at endometrial or intestinal mucosal surfaces (Yin et al., 2014; Forbester et al., 2015; Dolat and Valdivia, 2021; Hovhannisyan et al., 2024).

**TABLE 3:** Mechanistic archetypes for intracellular combination therapy.

Combination archetype	Intended complementarity	Preferred validation	Principal translational risk
Replicating-state drug + persister-active drug	Covers rapidly growing organisms and slow/nonreplicating survivors	State-resolved time-kill, washout, and relapse assays	Antagonism if one component suppresses growth required by the other
Penetrating drug + compartment-trapped drug	Covers spatially distinct cytosolic, vacuolar, or lesion populations	Co-localization imaging and compartment concentration–response	Functional monotherapy if neither drug reaches adequate exposure in other’s reservoir
Antibiotic + host-trafficking modulator	Improves phagosome maturation, autophagy, or target delivery	Host target-engagement marker with viable bacterial counts	Host toxicity or infection-stage-dependent immune effects
Antibiotic + metabolic restriction	Reduces nutrient supply while blocking stress survival/compensation	Isotope tracing, paired perturbation, and dynamic killing	Nutrient limitation may deepen tolerance instead of causing death
Antibiotic + resistance suppressor	Blocks efflux, repair, or emergence of resistant subpopulations	Mutation-frequency, regrowth, and serial-passage experiments	Pharmacokinetic interaction or off-target inhibition of host transporters
Sequential immune and antibacterial therapy	Uses dosing order to create a transient sensitized niche	Order-of-addition matrix under human-like concentration profiles	Incorrect timing may suppress protective immunity or miss sensitization window

These systems can deliver concentration–time profiles that mimic free plasma or tissue exposure. Sampling from separate vascular and epithelial channels, combined with live imaging and mass spectrometry, permits simultaneous estimation of tissue penetration, cellular uptake, bacterial killing, and host injury.

### 7.3 Microfluidic organ-on-a-chip: Specialized dynamic interrogation versus engineering bottlenecks

Microfluidic organ-on-a-chip (OOC) systems must not be misconstrued as broad-scale screening tools. Rather, they serve as low-throughput, highly specialized physical validation checkpoints for a strictly prioritized cohort of computationally nominated drug regimens. Their unique utility lies in recreating dynamic physical forces (e.g., alveolar stretch, microvascular shear stress, mucosal fluid flow) and complex multi-cellular architectures that static two-dimensional (2D) cultures completely fail to capture.

However, significant engineering and material constraints govern their translational pharmacokinetic fidelity.

### 7.4 Non-specific drug adsorption (The PDMS problem)

The pervasive use of poly (dimethylsiloxane) (PDMS) in microfluidic fabrication poses a major obstacle for hydrophobic, lipophilic small-molecule antimicrobials (e.g., bedaquiline, clofazimine, rifampicin). PDMS rapidly absorbs lipophilic compounds, leading to severe depletion of free unbound drug in the microfluidic channel and distorting local PK/PD metrics. Mitigating these artifacts requires either surface-passivation coatings, mathematical correction for sorption kinetics, or the adoption of inert, low-sorbing thermoplastic fluoropolymers (such as cyclic olefin copolymers [COC] or fluorinated ethylene propylene [FEP]).

### 7.5 Throughput and fluidic manifold complexity

OOC platforms operate at low throughput due to intricate fluidic pump systems, bubble-trap requirements, and complex tubing interfaces. Consequently, their role in the systems pharmacology pipeline is not discovery or primary combinatorial testing, but rather late-stage kinetic verification, validating the bactericidal clearance, persister suppression, and tissue-protective safety of top-tier regimens under dynamic, time-varying human exposure waveforms.

### 7.6 Minimum validation standards

A biomimetic infection model should document cell provenance, differentiation markers, barrier function, multiplicity of infection, extracellular-bacteria removal, bacterial localization, oxygen and pH, drug-binding conditions, and reproducibility across donors or devices. Pharmacological validation should include compounds with known high and low penetration, inactive structural controls, and clinically relevant unbound exposures.

For combination studies, essential outputs are viable bacterial burden, persister frequency, resistance emergence, host-cell death, barrier integrity, cytokines, and compartment-specific drug concentration. Reporting only fluorescence intensity is inadequate because reporter signal may persist after bacterial death or vary with metabolic state (Table 4).

**TABLE 4:** Proposed stage-gated workflow for Sub-PK/PD-guided combination development.

Stage	Core question	Preferred evidence	Advance criterion
Target mapping	Which host–pathogen dependency is active in the niche?	Dual/single-cell RNA-seq, metabolomics, isotope tracing, genetics	Causal dependency with a measurable biomarker
Exposure mapping	Do active molecules reach the bacterium?	Organelle fractionation, imaging MS, free intracellular concentration	Reproducible niche exposure above state-specific effective level
Combination testing	Are effects complementary over time?	Time-kill matrices, sequential dosing, persister and regrowth assays	Improved kill or relapse prevention without host toxicity
Computational integration	Which doses and schedules merit testing?	Mechanistic PK/PD, flux models, graph learning with uncertainty	Prospective prediction in held-out conditions
Biomimetic validation	Does activity persist in human-like tissue dynamics?	Primary cells, organoids, 3D cultures, organ-on-a-chip	Efficacy under realistic exposure profiles across donors
Translational qualification	Can target engagement and safety be monitored?	Biomarkers, interaction studies, population PK, resistance surveillance	Defined clinical hypothesis, dose rationale, and stopping rules

## 8 Discussion

### 8.1 Principal findings

The principal finding of this review is that intracellular antibacterial activity cannot be predicted reliably from extracellular potency or systemic exposure alone. The therapeutically relevant unit is a concentration–time profile acting on a defined bacterial state within a defined host compartment. This formulation incorporates three sources of heterogeneity that are often separated in conventional development: variation in drug distribution, variation in bacterial physiology, and variation in host-cell state. Treatment failure may arise from any one of these factors or from their interaction.

The available spatial pharmacology studies demonstrate why this distinction matters. Drugs with adequate plasma exposure can distribute unevenly between cellular lesion rims, necrotic material, immune-cell populations, and subcellular compartments (Prideaux et al., 2015; Blanc et al., 2018). A high total intracellular concentration may be pharmacologically useful when sequestration occurs beside the pathogen and the compound remains active, but misleading when the drug is trapped in an uninfected organelle, extensively membrane-bound, or chemically impaired by local pH (Schump et al., 2017; Greenwood et al., 2019). Sub-PK/PD therefore reframes intracellular penetration as a quantitative localization problem rather than a binary property of being “cell permeable.”

A second major finding is that pathogen location and bacterial physiological state must be considered simultaneously. Bacteria occupying the same nominal host-cell type can reside in phagosomes of different maturity, the host cytosol, lipid-associated structures, or damaged vacuoles. Within those sites, organisms may replicate rapidly, grow slowly, or enter nonreplicating persister states. Macrophage-induced efflux in mycobacteria and intracellular persister formation in *Salmonella* illustrate how host entry can change antibiotic susceptibility without producing stable genetic resistance (Adams et al., 2011; Helaine et al., 2014; Stapels et al., 2018). Consequently, an exposure that is sufficient for actively growing organisms may leave a metabolically altered reservoir intact.

The third finding is that host-directed and pathogen-directed therapy should not be conceptualized as separate development strategies. Intracellular pathogens depend on host trafficking, metabolism, and immune signaling, while these same processes determine drug delivery and bacterial stress. Host modulation may increase antibacterial activity by improving phagosome maturation, limiting access to a critical nutrient, enhancing autophagy, or preventing an inflammatory state that damages tissue without clearing infection (Stanley et al., 2014; Parihar et al., 2014; Singhal et al., 2014; Mayer-Barber et al., 2014). The strongest rationale for a host-directed partner is therefore not general immune stimulation, but a defined mechanism that complements the antibacterial drug in the relevant niche.

Finally, the review identifies a translational sequence that connects discovery technologies. Spatial pharmacology determines whether the active compound reaches the target site. Dual transcriptomics, single-cell profiling, and isotope tracing identify the host and bacterial processes that support survival at that site. Mechanistic and data-driven models prioritize combinations and schedules. Biomimetic systems then test those predictions under tissue architecture and fluctuating exposure. No single method is sufficient, but their integration can convert a descriptive observation into a testable treatment hypothesis.

### 8.2 Interpretation of the sub-PK/PD framework

Conventional PK/PD links a systemic exposure metric, such as the unbound area under the curve or time above the minimum inhibitory concentration, to a microbiological outcome. This approach remains essential, but intracellular infection inserts additional transfer functions between plasma and the organism. Drug must penetrate the lesion, enter the relevant host cell, partition into the pathogen-containing compartment, remain unbound and chemically active, cross the bacterial envelope, and engage its target. Loss at any step can uncouple plasma exposure from bacterial killing.

Sub-PK/PD does not require a separate mathematical model for every organelle. Its practical value is to identify which intermediate exposure measurement explains an observed discrepancy. For example, when plasma and tissue exposure are adequate but intracellular activity is poor, the next informative measurements are cellular uptake, organelle localization, free intracellular fraction, and activity under matched pH and nutrient conditions. When early killing is followed by regrowth, the priority shifts toward bacterial-state distributions, drug washout kinetics, and persister survival. This staged use of measurements can reduce unnecessary experimentation while preserving mechanistic resolution.

The framework also highlights that subcellular accumulation can be beneficial, neutral, or harmful. Lysosomal trapping may generate a local reservoir that prolongs exposure, but it can also lower free cytosolic concentration or expose host organelles to toxicity. Lipid binding may facilitate delivery to lipid-rich infected macrophages yet reduce the immediately diffusible fraction. The correct interpretation depends on co-localization, exchange kinetics, chemical stability, and target engagement. Total drug concentration should therefore be accompanied, where feasible, by an estimate of the active unbound fraction and a spatial measurement that confirms proximity to bacteria.

An important conceptual extension is to treat the intracellular niche as dynamic. Vacuoles mature, membranes become damaged or repaired, host cells change metabolic state, and bacteria move between replicating and nonreplicating phenotypes. Drug localization measured at one time point may not represent exposure later in treatment. Longitudinal imaging, pulse–chase experiments, and compartmental models are needed to determine whether a drug follows the pathogen as the niche changes or whether a period of spatial mismatch emerges.

### 8.3 Meaning of synergy in an intracellular system

Synergy is frequently defined from a checkerboard assay conducted at constant concentrations in nutrient-rich medium. Such measurements are useful for initial screening but do not establish intracellular therapeutic complementarity. In a host cell, an apparent interaction can arise through several distinct mechanisms: direct interaction at bacterial targets, improved penetration of one drug by another, host-mediated restriction of nutrients, altered bacterial state, or prevention of regrowth from a persister population. Distinguishing these mechanisms matters because they imply different dose ratios and schedules.

Spatial co-exposure is a necessary consideration. If one component accumulates in lysosomes while the other remains largely cytosolic, the combination may not act on the same organisms. This can still be useful if two spatially distinct reservoirs contribute to persistence, but the therapeutic objective is then population coverage rather than molecular synergy. Conversely, two compounds may reach the same niche but at different times because of distinct absorption, distribution, or organelle-release kinetics. The interval during which only one drug is active creates functional monotherapy and may undermine resistance suppression.

Temporal sequence is especially important for combinations involving host metabolism or immunity. A host-directed agent that accelerates phagosome maturation may need to precede the antibiotic, while a nutrient-restriction strategy could sensitize bacteria briefly before inducing a deeper tolerant state. An anti-inflammatory intervention introduced too early might suppress a protective response, whereas the same intervention later could reduce tissue damage. These possibilities cannot be resolved by simultaneous fixed-dose assays. Sequential dosing, repeated exposure, washout, and relapse experiments should be incorporated early in development.

The most informative endpoint is likewise context dependent. A reduction in intracellular luminescence may represent killing, growth arrest, or altered reporter activity. Viable counts, time to positivity, live/dead imaging, and regrowth after drug removal provide stronger evidence. For combinations intended to prevent resistance, the frequency and genotype of surviving colonies are relevant. For host-directed therapy, efficacy must be interpreted together with host-cell viability, barrier function, inflammatory signaling, and evidence of the intended host target engagement.

### 8.4 Integration of host–pathogen systems biology

Paired analysis of host and pathogen offers a route from correlation to rational combination design. Dual RNA sequencing can reveal coordinated induction of a host nutrient-supply pathway and a bacterial uptake or catabolic pathway, while single-cell methods can identify whether that dependency is concentrated in the cells carrying the highest bacterial burden (Westermann et al., 2016; Avraham et al., 2015; Saliba et al., 2016). These associations nominate candidate interfaces but do not establish metabolic flux or essentiality.

Flux analysis provides the next level of evidence. Stable-isotope tracing can determine which host-derived substrates enter bacterial metabolism, and constraint-based models can test whether alternative pathways compensate when one exchange route is blocked. The finding that intracellular *M. tuberculosis* uses a mixed carbon diet illustrates why expression data or single-substrate culture may underestimate metabolic flexibility (Beste et al., 2013). For therapy design, the most attractive vulnerability is not necessarily the pathway with the largest transcriptional change, but the pathway whose inhibition cannot be bypassed in the relevant niche.

This systems perspective also helps avoid counterproductive host interventions. Restricting one nutrient may force bacteria toward a slower, more drug-tolerant state or toward an alternative substrate abundant in the same compartment. A coupled model can predict these adaptations and suggest a second intervention that blocks the compensatory route or targets maintenance metabolism. Experimental validation should then test host and bacterial perturbations individually and in combination, using isotope tracing to confirm that the intended flux has changed.

### 8.5 Translational and clinical significance

For preclinical development, the framework supports a niche-specific target product profile. In addition to organism, indication, route, and systemic exposure, the profile should specify the principal infected cell type, subcellular compartment, bacterial state, desired local exposure, companion mechanism, and dosing sequence. This information determines which assays are decision-relevant. A compound intended for cytosolic *Listeria*, for example, should not be advanced solely on the basis of total macrophage accumulation, while a therapy for vacuolar organisms should demonstrate activity under the pH and nutrient conditions of that compartment.

Clinical translation will require biomarkers at more than one scale. Plasma pharmacokinetics alone may be insufficient when lesion or cellular penetration is highly variable. Tissue imaging, bronchoalveolar or lesion sampling, circulating host-response signatures, and pathogen-state biomarkers could help determine whether failure reflects inadequate exposure, absent target engagement, or biological nonresponse. These measurements need not be applied to every patient, but early-phase studies should establish whether systemic dose predicts the relevant local effect.

Combination selection should also account for patient heterogeneity. Diabetes, lipid disorders, immunosuppression, renal or hepatic dysfunction, and concomitant medicines can alter host metabolism and drug distribution. The safety and efficacy of a host-directed component may therefore vary more strongly across patients than that of a conventional antibacterial. Biomarker-guided enrichment may be appropriate when the targeted host pathway is active only in a subset of infections.

The framework may also improve antimicrobial stewardship. Regimens can be designed to minimize periods of spatial or temporal monotherapy, and development programs can identify whether relapse is driven by genetic resistance or by a protected phenotypic reservoir. This distinction affects the response: resistance may require a new antibacterial mechanism, whereas persistence may be better addressed by improved penetration, prolonged niche exposure, or a partner active against nonreplicating bacteria.

### 8.6 Strengths and limitations of the evidence

The major strength of the evidence is methodological convergence. Spatial imaging and chemical analysis show heterogeneous drug distribution; genetic and pharmacological perturbations demonstrate host contributions to intracellular survival; paired transcriptomics and isotope tracing reveal cross-species metabolic dependencies; and organ-on-a-chip studies show that tissue architecture changes infection behavior (Prideaux et al., 2015; Westermann et al., 2016; Thacker et al., 2020; Sharma et al., 2021). Similar conclusions emerging from different technologies increase confidence in the central premise that local context governs efficacy.

The evidence also contains strong mechanistic examples rather than associations alone. Cholesterol utilization is required for mycobacterial persistence, host-targeted screens identify pathways that restrict intracellular growth, and experimental host modulation can enhance antimicrobial control (Pandey and Sassetti, 2008; Stanley et al., 2014; Parihar et al., 2014). Computational approaches have progressed beyond generic compound ranking by predicting drug synergy and linking interaction models to pharmacokinetics and granuloma behavior (Ma et al., 2019; Cicchese et al., 2021). These studies demonstrate the feasibility of integrating scales, even though routine implementation remains limited.

Several limitations constrain generalization. Tuberculosis and *Salmonella* dominate the literature, while obligate intracellular bacteria and uncommon clinical pathogens are less well represented. Results derived from macrophages may not transfer to epithelial cells, hepatocytes, osteoblasts, or endothelial cells. Laboratory strains may also differ from multidrug-resistant clinical isolates in transporter expression, metabolic flexibility, and stress adaptation.

Experimental definitions are inconsistent. “Intracellular activity” may refer to reduced fluorescence, lower total bacterial DNA, inhibition of replication, or loss of culturable organisms. Drug exposure is reported as nominal medium concentration, total-cell concentration, or tissue abundance, often without protein binding or unbound fraction. These differences complicate comparison and prevent quantitative synthesis. Standardized reporting of exposure, viable burden, host toxicity, and post-treatment regrowth would materially improve the field.

Each measurement technology has additional biases. Subcellular fractionation can disrupt organelles and cause cross-contamination. Fluorescent modification may alter the permeability, charge, binding, or activity of a drug. Imaging mass spectrometry provides spatial information but may lack single-organelle resolution and generally measures total analyte. Omics methods face low bacterial biomass, dropout, and difficulty distinguishing cause from response. Isotope tracing depends on model structure and substrate labeling, while flux balance analysis inherits incomplete annotations and assumptions about cellular objectives.

Computational and AI studies are limited by data quality and domain shift. Training sets contain repeated scaffolds, laboratory conditions, and a small number of species, creating a risk of optimistic performance when validation samples resemble training data. Combination datasets are especially sparse because dose ratio, timing, medium, host cell, and endpoint all influence the interaction label. Models should therefore be judged by prospective performance in held-out biological contexts, calibration of uncertainty, and experimental confirmation rather than internal cross-validation alone.

Biomimetic models provide greater physiological realism but introduce lower throughput, technical variability, and challenges in validating which aspects of human disease they reproduce. A microfluidic device can model flow and tissue interfaces without reproducing adaptive immunity, organ-level metabolism, or long-term disease. These systems should be used as intermediate evidence rather than assumed replacements for animal studies or clinical investigation.

Finally, this review is a targeted narrative synthesis. Its iterative search and concept-driven selection may have missed relevant reports and give greater prominence to well-characterized examples. The proposed framework is therefore best understood as an organizing model that generates testable hypotheses, not as evidence that any specific combination is clinically superior.

### 8.7 Implications for practice and development

The framework does not imply that every program requires exhaustive organelle pharmacology. Measurements should be selected according to the dominant uncertainty and the cost of making a wrong development decision. If potent intracellular activity *in vitro* fails to translate *in vivo*, lesion penetration, protein binding, and exposure in the infected cell type should be examined. If total-cell exposure is high but killing is weak, localization, free fraction, pH-conditioned potency, and bacterial state become priorities. If initial killing is followed by relapse, persister frequency, drug-release kinetics, and the duration of joint combination coverage should be measured.

A stage-gated workflow can make this approach operational. Early discovery should establish direct antibacterial potency, host toxicity, and activity in at least one relevant infected-cell model. Lead optimization should then examine the physicochemical determinants of niche exposure. Combination development should use dynamic time-kill and sequential-dosing studies rather than checkerboards alone. Advanced candidates should be tested in primary cells or organoids and then in a microphysiological or animal model capable of reproducing the relevant tissue barrier and dosing profile.

Go/no-go criteria should be mechanistically explicit. A host-directed candidate should demonstrate target engagement, an acceptable therapeutic window, and added value over the antibiotic backbone. A computationally proposed combination should reproduce its predicted interaction prospectively and remain effective across plausible dose ratios. A repurposed compound should achieve a clinically attainable unbound concentration at the target compartment rather than merely show activity at a nominal screening concentration.

Regulatory and clinical development strategies will also need to consider how the contribution of each component is demonstrated. Factorial or adaptive early-phase designs may help distinguish antibacterial, host-directed, and interaction effects. Safety monitoring should reflect the host pathway being modulated, while microbiological endpoints should include relapse or sustained clearance when persistence is the proposed target. Mechanistic biomarkers can reduce ambiguity when a regimen fails and guide dose or schedule refinement.

### 8.8 Implications for research

Several methodological gaps are tractable:

1. The field needs reference compounds and shared protocols for measuring total and unbound intracellular drug, validating organelle fractionation, and relating spatial signal to viable bacterial burden. Reference agents with known cytosolic, lysosomal, membrane, and extracellular distribution would allow laboratories to compare platform performance.
2. Intracellular PK/PD datasets should report common metadata: pathogen strain, host-cell source and activation state, multiplicity and duration of infection, localization method, medium composition, protein binding, exposure waveform, viable burden, host toxicity, and regrowth after washout. Public release of negative and antagonistic combination results would reduce duplication and improve computational training data.
3. Coupled host–pathogen models require systematic experimental benchmarking. Predictions of nutrient exchange should be tested with isotope tracing and targeted genetic perturbation in both organisms. Models should represent maintenance and stress-survival objectives rather than bacterial biomass alone, particularly when studying nonreplicating populations.
4. Single-cell and spatial methods should be connected directly to pharmacology. Measuring bacterial state and host response in the same cells in which drug localization is quantified would reveal whether treatment failure is caused by low exposure, intrinsic tolerance, or both. Longitudinal approaches are particularly important because surviving populations after treatment may differ substantially from the pretreatment average.
5. Biomimetic models should incorporate clinically realistic free-drug concentration profiles and report variability across donors and devices. Inter-laboratory comparisons are needed to establish reproducibility, and model qualification should be indication specific. A bladder-chip, granuloma model, and alveolar interface reproduce different barriers and should not be treated as generic “advanced” models.
6. The most valuable future studies will be prospective integration studies. Rather than applying each technology retrospectively, investigators should begin with a defined intracellular failure mechanism, predict a combination and schedule, measure target-site exposure, test the predicted metabolic or immune effect, and validate sustained killing in a biomimetic model. This closed-loop design would provide the strongest evidence that systems pharmacology and Sub-PK/PD improve therapeutic decisions.

### 8.9 Ethical, practical, and implementation considerations

#### 8.9.1 Safety of host-directed therapy

Manipulating host metabolism or immunity introduces risks not captured by bacterial selectivity assays. The same pathway may be protective early in infection and damaging later, or may differ between lung, liver, and bone marrow. Immunocompromised patients, pregnant individuals, children, and people with metabolic disease may have narrower safety margins. Development should therefore include cell-type-resolved toxicity, infection-stage biomarkers, interaction testing, and clear criteria for discontinuing the host-directed component.

#### 8.9.2 Responsible use of artificial intelligence

Training data for antibiotic activity are concentrated in a few species, laboratory strains, and assay formats. Models may consequently underperform against rare pathogens, resistant clinical isolates, or host environments absent from the training set. Data provenance, assay harmonization, external validation, uncertainty estimates, and interpretable rationales are necessary before AI-ranked combinations influence clinical decisions. Predictions should prioritize experiments, not substitute for microbiological and pharmacological validation.

#### 8.9.3 Equity, access, and antimicrobial stewardship

Complex regimens and microphysiological testing can increase development cost. A therapy that requires expensive diagnostics, cold-chain biologics, or intensive monitoring may be inaccessible where intracellular infections are most prevalent. Target product profiles should include affordability, stability, route of administration, and compatibility with decentralized care from the outset.

Combination therapy also has stewardship implications. Poor adherence or mismatched half-lives can create periods of effective monotherapy. Regimens should be designed for forgiveness, with compatible pharmacokinetics and resistance barriers. Surveillance should distinguish phenotypic persistence from genetic resistance while monitoring both.

#### 8.9.4 Future directions and recommendations

1. Intracellular drug development should routinely measure unbound, compartment-specific exposure rather than relying on plasma or total-cell concentration. Spatial measurements need not be universal, but they should be triggered whenever efficacy and systemic exposure diverge.
2. Combination screens should reproduce the physicochemical and metabolic state of the target niche. At minimum, this includes relevant pH, oxygen, nutrient composition, cell type, and dynamic drug exposure. Time-kill, washout, and regrowth experiments should replace single endpoint synergy as the principal decision tools.
3. Cross-species target discovery should integrate dual transcriptomics with flux measurements. Candidate bottlenecks should be validated by perturbing both the host supply pathway and the bacterial utilization pathway, alone and with a direct antibacterial.
4. Predictive models should combine molecular structure with compartment, host state, bacterial state, and pharmacokinetics. Prospective evaluation should use held-out pathogens, cell types, and schedules. Model uncertainty should determine which experiments are most informative, enabling active-learning cycles.
5. Advanced human models should be used as stage gates before animal-intensive or clinical studies. Organoids and organ-on-a-chip systems are particularly valuable for comparing dosing order, testing donor heterogeneity, and identifying host toxicity that simple monolayers miss. Their adoption requires shared validation standards and inter-laboratory reproducibility studies.
6. Clinical translation should be biomarker-led. Trials of host-directed partners should confirm the intended host effect, while antibiotic components should be supported by lesion or intracellular exposure models. Adaptive designs may be useful when response depends on host phenotype or pathogen niche.

## 9 Conclusion

Intracellular bacterial persistence arises from the interaction of anatomical barriers, subcellular drug partitioning, bacterial state, and host metabolic and immune remodeling. Treating these infections as homogeneous extracellular systems obscures the mechanisms that cause therapeutic failure. A Sub-PK/PD framework makes the relevant unit of analysis explicit: active drug at the bacterial niche over time.

When combined with dual host–pathogen profiling, metabolic flux analysis, interpretable computational prediction, and biomimetic validation, Sub-PK/PD can transform combination design from empirical pairing into mechanism-based regimen construction. The most promising regimens will not merely contain multiple active agents; they will deliver complementary activities to the correct compartments, against the relevant bacterial states, in a sequence that preserves host function and suppresses resistance.
